# Anti-Photoaging and Anti-Melanogenic Effects of a Novel *Lactiplantibacillus plantarum* RKBP Isolated from Giant *Centella asiatica*

**DOI:** 10.4014/jmb.2603.03011

**Published:** 2026-06-01

**Authors:** Jiwon Seo, Eun Jin Sung, Do Yeong Son, Dae Sung Yoo, Dae Bang Seo, Myunghwa Kim, Chan Yoo

**Affiliations:** 1 Advanced Technology R&D Institute, ASK LABS, 05830, Seoul, Republic of Korea; 2 ASK COMPANY Co., Ltd., 41256, Daegu, Republic of Korea; 3 Department of Biocosmetic, Sungkyunkwan University, Suwon 16419, Republic of Korea; 4 Department of Molecular and Genetic Engineering, Korea University, Seoul 02841, Republic of Korea

**Keywords:** *L. plantarum* RKBP, Probiotics, Anti-photoaging, Anti-melanogenesis, Skin health

## Abstract

Ultraviolet B (UVB) irradiation is a major contributor to photoaging and hyperpigmentation, leading to collagen damage and excessive melanin accumulation. This study investigated the anti-photoaging and anti-melanogenic effects of heat-killed *L. plantarum* RKBP isolated from Giant *Centella asiatica*. *L. plantarum* RKBP was prepared by thermal inactivation and evaluated for anti-photoaging effects using human dermal fibroblasts and three-dimensional (3D) human skin-equivalent tissues. The anti-melanogenic effects were assessed in α-melanocyte-stimulating hormone (α-MSH)-stimulated B16F10 melanoma cells, three-dimensional melanoma spheroids, and intrinsically pigmented 3D human skin-equivalent models. MAPK/AP-1 signaling, MMP-1 expression, collagen integrity, melanin content, and melanogenesis-related protein expression were analyzed. *L. plantarum* RKBP selectively suppressed UVB-induced ERK/JNK phosphorylation and AP-1-mediated MMP-1 expression, preserving collagen integrity in a 3D human skin-equivalent model. Treatment with *L. plantarum* RKBP reduced α-MSH-induced melanin production and decreased tyrosinase expression in B16F10 cells. *L. plantarum* RKBP also attenuated melanin accumulation in 3D melanoma spheroids and pigmented human skin-equivalent tissues. *L. plantarum* RKBP exerts anti-photoaging and anti-melanogenic effects across *in vitro* and 3D skin models. These findings support its potential as a postbiotic skin health ingredient.

## Introduction

Exposure to ultraviolet B (UVB) radiation induces visible signs of aging skin, such as wrinkles, loss of elasticity, and uneven pigmentation [1]. To understand these effects, it is essential to consider skin architecture. Human skin is composed of the epidermis, dermis, and subcutaneous tissue [2]. Among these layers, the dermis plays a key role in maintaining structural integrity. The dermis, primarily composed of Type I collagen, maintains skin elasticity and biomechanical stability. Disruption of collagen homeostasis, particularly the balance between its synthesis and degradation, is a critical mechanism driving the photoaging process [3].

UV radiation is divided into UVA (320-400 nm), UVB (280-320 nm), and UVC (200-280 nm). UVB has the highest biological impact despite limited skin penetration [4]. UVB exposure induces the generation of reactive oxygen species (ROS), leading to oxidative stress and upregulation of pro-inflammatory cytokines. This cascade activates the MAPK (ERK, JNK, p38)/AP-1 signaling pathway, which stimulates the expression of matrix metalloproteinases (MMPs), including MMP-1, MMP-3, and MMP-9 [5]. These enzymes degrade dermal collagen and ultimately result in wrinkle formation and decreased skin elasticity [6]. Consequently, suppressing MMP activity and maintaining collagen integrity represent critical strategies in photoaging prevention.

Beyond these structural effects, UVB exposure triggers melanogenesis as a protective mechanism. UVB irradiation stimulates α-melanocyte-stimulating hormone (α-MSH) secretion from keratinocytes. α-MSH binds to melanocortin-1 receptor (MC1R) on melanocytes. This activation leads to increased intracellular cAMP levels, subsequently activating the PKA-CREB-MITF signaling cascade and upregulating tyrosinase expression [7]. While melanin production serves as a natural photoprotective response, excessive or uneven melanogenesis can result in hyperpigmentation disorders, age spots, and melasma [8].

Recent studies have demonstrated that probiotics can modulate immune responses and influence skin health. For instance, *L. plantarum* HY7714 improved skin health by reducing matrix metalloproteinases (MMPs) and other inflammatory markers [9]. Additionally, *L. plantarum* CJLP133 alleviated atopic dermatitis in clinical studies [10]. These findings suggest that specific probiotic strains can influence skin homeostasis through various mechanisms.

In this study, we focused on *Lactiplantibacillus plantarum* RK BYoungPool (RKBP, KCTC 14901BP), a plant-derived lactic acid bacterium that was isolated from the leaves of Giant *Centella asiatica* (GCA), a medicinal herb traditionally used for skin health [11]. Our previous study demonstrated that *L. plantarum* RKBP exhibits excellent safety profiles and probiotic characteristics, including superior intestinal adhesion ability (45% higher than control) and enhanced expression of tight junction-related genes (ZO-2, ZO-3, occludin, and claudin-2) [12]. In addition, this strain is registered as a notified probiotic strain by the Korean Ministry of Food and Drug Safety, supporting its regulatory reliability for application in functional products. Notably, the source plant, GCA, has been shown to exhibit superior anti-melanogenic effects compared to conventional *Centella asiatica* [7]. This enhanced activity is attributed to its higher content of madecassoside and asiaticoside, which directly bind to MC1R. Based on these observations, we hypothesized that *L. plantarum* RKBP isolated from GCA may also possess skin-beneficial properties. Taking together, these characteristics suggest that *L. plantarum* RKBP is a promising plant-derived probiotic candidate for skin-related applications. Therefore, this study investigated the dual anti-photoaging and anti-melanogenic effects of *L. plantarum* RKBP in comparison with *L. plantarum* ATCC 14917, used as a reference strain. We evaluated the protective effects of *L. plantarum* RKBP against UVB-induced MMP-1 upregulation and collagen loss, as well as against α-MSH-induced melanogenesis, using both *in vitro* cellular models and 3D human skin-equivalent models.

## Materials and Methods

### Preparation of *L. plantarum* Strains

*L. plantarum* RKBP and *L. plantarum* type strain (ATCC 14917, referred to as “Type” in figures) were cultured in MRS broth from Becton Dickinson (USA) at 37°C for 16 h. The precultured strains were inoculated into 1 L of MRS broth at an initial optical density (OD) of 0.1 and incubated under the same conditions. Cells were harvested by centrifugation at 6,837 × g for 20 min at 4°C, washed twice with PBS, and resuspended in PBS. The bacterial suspension was adjusted to 1 × 10^11^ CFU/mL and heat-treated at 90°C for 30 min to obtain heat-killed preparations, which were stored at 4°C until use.

### Reagents

Dulbecco's Modified Eagle Medium (DMEM) and fetal bovine serum (FBS) were purchased from Hyclone (USA) and Seradigm (USA), respectively. As positive controls, retinol and arbutin were purchased from Sigma-Aldrich (USA). Retinol was used at 5 μM for anti-photoaging experiments, while arbutin was applied at 100 μg/mL for anti-melanogenic experiments, concentrations previously shown to be effective without causing cytotoxicity [7, 13]. α-MSH was obtained from Sigma-Aldrich. Primary antibodies against MMP-1, β-actin, tyrosinase, ERK, p-ERK, JNK, p-JNK, p38, p-p38, and MITF were used in this study. Details of all primary antibodies, including supplier, catalog number, and dilution, are provided in Table 1.

### Cell Culture and UVB Irradiation

Human dermal fibroblasts (HDFs) were cultured in DMEM containing 10% FBS at 37°C with 5% CO_2_. Cells were seeded in 6-well plates (2 × 10^4^ cells/well) and incubated for 24 h. The culture medium was exchanged with serum-free DMEM, and cells were treated with heat-killed RKBP or ATCC 14917 (0.5 to 2 × 10^8^ CFU/mL) for 1 h before UVB exposure. A Bio-Link irradiation system (Paris, France) was used for UVB irradiation at 0.01 J/cm^2^. All tested concentrations showed no cytotoxicity as confirmed by MTT assay (data not shown).

### Western Blot

Protein expression was analyzed by western blotting as described previously [14]. After UVB exposure, cells were lysed using RIPA buffer from Cell Signaling Technology (USA), and protein concentrations were measured using a BCA assay kit from Pierce Biotechnology (USA). Equal amounts of protein were resolved on SDS-PAGE gels and transferred onto PVDF membranes. Membranes were blocked, incubated with primary antibodies, followed by HRP-conjugated secondary antibodies. Protein bands were visualized using a chemiluminescence imaging system from e-BLOT (China). Band intensities were quantified using ImageJ software (version 1.52v) from National Institutes of Health (USA).

### Luciferase Reporter Gene Assay

The construction of the MMP-1 promoter luciferase vectors and lentiviral transduction procedures followed our previously published protocol [15]. Lentiviral particles were generated and used to transduce HaCaT cells, and successfully transduced cells were selected with puromycin as described earlier [14, 16]. For the luciferase assay, transduced HaCaT cells were cultured in 6-well plates (4 × 10^5^ cells/well) and switched to serum-free DMEM. The cells were pretreated with test samples for 1 h and then exposed to UVB (0.01 J/cm^2^). After 6 h, the cells were harvested, and AP-1 transactivation was quantified using a luciferase assay kit from Promega (USA).

### 3D Human Skin-Equivalent Model

The 3D human skin-equivalent model (Neoderm^®^-ED) from Tego Science (Republic of Korea) was used as previously described [14]. Photoaging was induced by exposing the tissues to UVB (0.01 J/cm^2^) twice daily for 4 days. 24 h after the final UVB irradiation, the culture media were collected. Total protein concentrations were quantified using a BCA assay, and equal amounts of protein were analyzed by western blotting to determine MMP-1 expression. The 3D human skin-equivalent models are commercially available reconstructed human skin tissues, and therefore ethical approval and informed consent were not required.

### Gelatin Zymography

MMP-2 was used as a loading control for MMP-1 western blots, because MMP-2 protein levels are not affected by UVB exposure [17]. Protein samples were separated using SDS-PAGE with gelatin, followed by incubation in renaturing and developing buffers. The gels were stained with Coomassie blue to visualize gelatinolytic activity.

### Masson's Trichrome Staining and Immunohistochemical Examination

Collagen content in the 3D human skin-equivalent model was assessed using Masson's trichrome staining as previously described [14]. After the final UVB exposure, tissues were fixed in 4% formaldehyde and processed for paraffin embedding and sectioning. Paraffin-embedded sections (3 μm) were stained with Masson's trichrome to evaluate collagen fibers. For immunohistochemical (IHC) analysis of MMP-1, sections were processed as previously described [18] and incubated with MMP-1 primary antibody, followed by an HRP polymer-based detection system with DAB chromogen. Slides were examined under an Olympus AX70 light microscope (Japan). Collagen density and MMP-1 immunostaining intensities were quantified using Adobe Photoshop software (version 26.11.2) from Adobe (USA).

### Measurement of Melanin Content

Melanin secretion was quantified in B16F10 murine melanoma cells as previously described [7]. Cells were seeded at a density of 2 × 10^5^ cells per 60-mm dish and incubated overnight. The cells were pre-treated with *L. plantarum* RKBP and ATCC 14917 (1 to 4 × 10^8^ CFU/mL) for 1 h and then stimulated with α-MSH (200 nM) for 72 h. The conditioned media were collected and centrifuged at 13,572 × g for 10 min, and the supernatants were transferred to a 96-well plate. Extracellular melanin levels were determined by measuring absorbance at 490 nm using an Epoch microplate reader (USA).

### A Three-Dimensional Melanoma Cell Culture System

A three-dimensional melanoma spheroid model was generated using the forced-floating method as previously described [7]. B16F10 cells (1 × 10^4^ cells/well) were seeded into ultra-low attachment (ULA) 96-well round plates and incubated overnight at 37°C. The spheroids were then co-treated with α-MSH (200 nM) and either *L. plantarum* RKBP (1-4 × 10^8^ CFU/mL), ATCC 14917 (4 × 10^8^ CFU/mL), or arbutin (100 μg/mL) and maintained for 3 days. Melanin levels in the spheroids were quantified by measuring absorbance at 490 nm.

### 3D Human Skin-Equivalent Model for Melanogenesis

The 3D human skin-equivalent model (Neoderm^®^-ME), composed of human epidermal keratinocytes and melanocytes, was used to assess pigmentation. The tissues were maintained in the manufacturer’s medium containing *L. plantarum* RKBP, ATCC 14917, or arbutin at 37°C and 5% CO_2_ for 7 days. The medium was replaced every 2 days. On day 7, pigmentation of the tissues was examined using an Olympus AX70 light microscope. The tissues were then lysed, centrifuged for 10 min at 13,572 × g, and analyzed for tyrosinase expression. Total protein concentrations were determined using a BCA assay according to the manufacturer’s protocol.

### Fontana-Masson Staining

Fontana-Masson staining was performed to visualize melanin in the 3D human skin-equivalent tissues as previously described [7]. The tissues were fixed in 4% formaldehyde, embedded in paraffin, and sectioned at 3 μm. The sections were processed using standard Fontana-Masson silver staining procedures and counterstained with nuclear fast red. The stained slides were then examined using an Olympus AX70 light microscope. Staining intensities were quantified using Adobe Photoshop software.

### Statistical Analyses

Statistical analyses were conducted using SPSS software version 12.0K (USA). Data are presented as the mean ± standard deviation. Comparisons between two groups were assessed using an unpaired Student’s t-test. For analyses involving more than two groups, one-way ANOVA followed by Tukey’s Honest Significant Difference (HSD) post-hoc test was performed. *P* < 0.05 was considered statistically significant.

## Results

### Suppression of UVB-Induced MMP-1 Expression

**Fig. 1:**
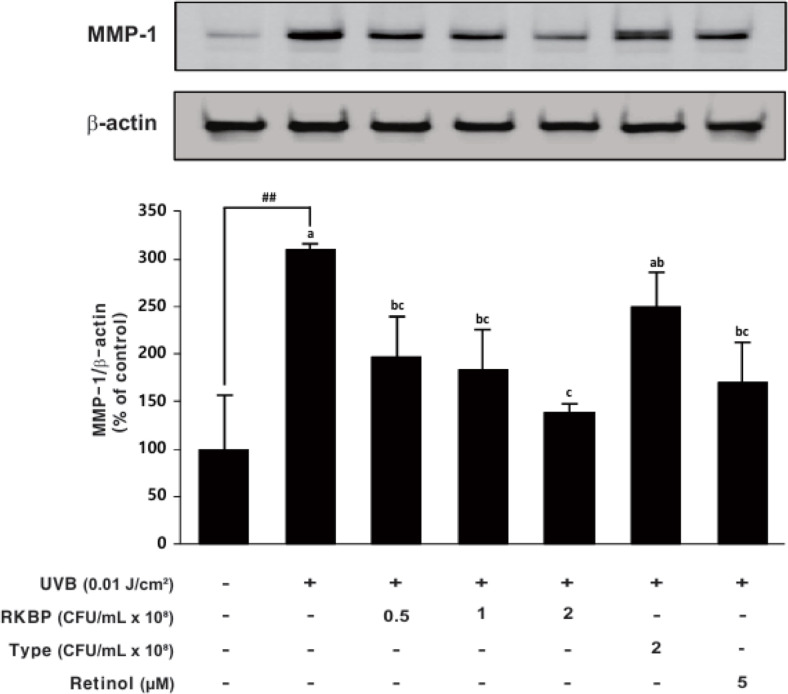
Inhibitory effects of *L. plantarum* RKBP on UVB-induced MMP-1 expression. Representative western blots of MMP-1 and β-actin are shown. MMP-1 levels were quantified after normalization to β-actin. Type indicates *L. plantarum* ATCC 14917. Data are presented as mean ± SD (n = 3). Significant differences between the untreated control and UVB-induced group (^##^*P* < 0.01). Different letters indicate statistically significant differences among the treatment groups based on one-way ANOVA followed by Tukey's HSD test (*P* < 0.05).

UVB irradiation of HDFs resulted in a more than three-fold increase in MMP-1 secretion compared with the control, confirming the successful induction of UVB-induced photoaging conditions (Fig. 1). Treatment with *L. plantarum* RKBP significantly attenuated this UVB-induced MMP-1 increase in a dose-dependent manner. *L. plantarum* RKBP reduced MMP-1 secretion by 27.3%, 33.6%, and 47.0% at concentrations of 0.5, 1, and 2 × 10^8^ CFU/mL, respectively. In contrast, ATCC 14917 reduced MMP-1 levels by 15.7% at 2 × 10^8^ CFU/mL under the same conditions. Retinol, used as a reference compound [19], also decreased UVB-induced MMP-1 levels. Collectively, these results demonstrate that *L. plantarum* RKBP exerts a stronger inhibitory effect on UVB-induced MMP-1 expression than ATCC 14917.

### *L. plantarum* RKBP Attenuates UVB-Induced AP-1 Transactivation

**Fig. 2:**
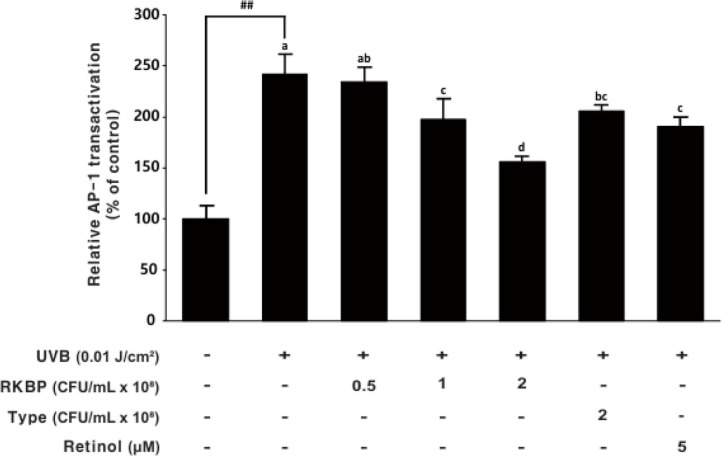
*L. plantarum* RKBP attenuates UVB-induced AP-1 transactivation. AP-1 luciferase activity was measured in HaCaT reporter cells after UVB irradiation and treatment with *L. plantarum* RKBP, ATCC 14917, or retinol. Type indicates *L. plantarum* ATCC 14917. Data are presented as mean ± SD (n = 3). Significant differences between the untreated control and UVB-induced group (^##^*P* < 0.01). Different letters indicate statistically significant differences among the treatment groups based on one-way ANOVA followed by Tukey’s HSD test (*P* < 0.05).

AP-1 is a key transcription factor that regulates UVB-induced MMP-1 expression. To evaluate whether *L. plantarum* RKBP modulates this

response, AP-1 luc HaCaT cells were exposed to UVB and subsequently treated with *L. plantarum* RKBP at various concentrations. UVB irradiation increased AP-1 transactivation to approximately 2.4-fold relative to the untreated control (Fig. 2). *L. plantarum* RKBP significantly suppressed UVB-induced AP-1 transactivation in a dose-dependent manner. At 2 × 10^8^ CFU/mL, *L. plantarum* RKBP reduced UVB-induced AP-1 transactivation by 35.5%, whereas ATCC 14917 exhibited a reduction of 15.3%. These findings indicate that *L. plantarum* RKBP exerts a stronger inhibitory effect on UVB-induced AP-1 transactivation than ATCC 14917.

### *L. plantarum* RKBP Attenuates UVB-Induced Phosphorylation of ERK and JNK

**Fig. 3:**
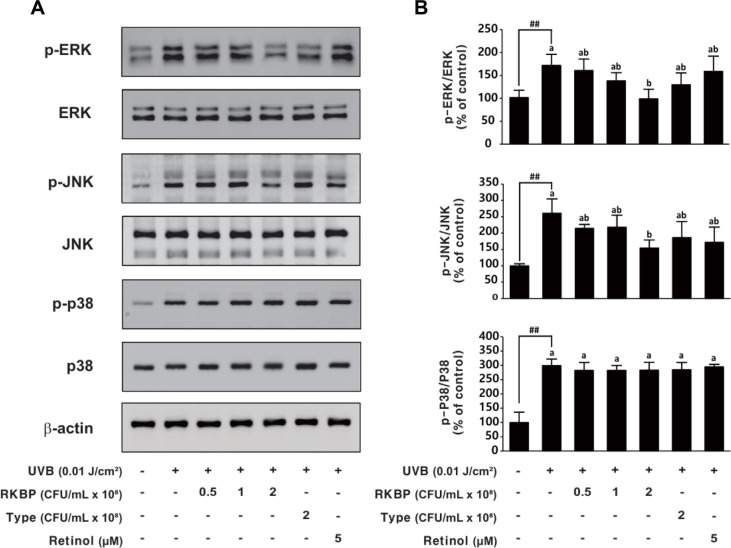
*L. plantarum* RKBP attenuates UVB-induced MAPK phosphorylation in human dermal fibroblasts. (**A**) Representative western blot bands showing phosphorylated and total ERK, JNK, and p38 in HDFs after UVB irradiation and treatment with *L. plantarum* RKBP or ATCC 14917. β-Actin was used as a loading control. (**B**) Quantification of phosphorylated-to-total protein ratios for ERK, JNK, and p38. Type indicates *L. plantarum* ATCC 14917. Data are presented as mean ± SD (n = 3). Significant differences between the untreated control and UVB-induced group (^##^*P* < 0.01). Different letters indicate statistically significant differences among the treatment groups based on one-way ANOVA followed by Tukey’s HSD test (*P* < 0.05).

UVB irradiation activates MAPK pathways, including ERK, JNK, and p38, which play key roles in regulating stress responses and photoaging-related signaling. Exposure of HDFs to UVB markedly increased the phosphorylation of ERK, JNK, and p38 compared with the untreated control. Treatment with *L. plantarum* RKBP significantly reduced ERK and JNK phosphorylation at 2 × 10^8^ CFU/mL (Fig. 3A), while showing no effect on p38 phosphorylation, indicating pathway selectivity. ATCC 14917 exhibited a weaker inhibitory effect on ERK and JNK phosphorylation relative to *L. plantarum* RKBP. The quantitative analysis of MAPK phosphorylation levels further confirmed these selective inhibitory effects (Fig. 3B). These findings suggest that *L. plantarum* RKBP selectively modulates the ERK and JNK pathways within the MAPK signaling cascade, thereby reducing UVB-induced MMP-1 expression.

### *L. plantarum* RKBP Attenuates UVB-Induced MMP-1 Overexpression and Collagen Downregulation in a 3D Human Skin-Equivalent Model

**Fig. 4:**
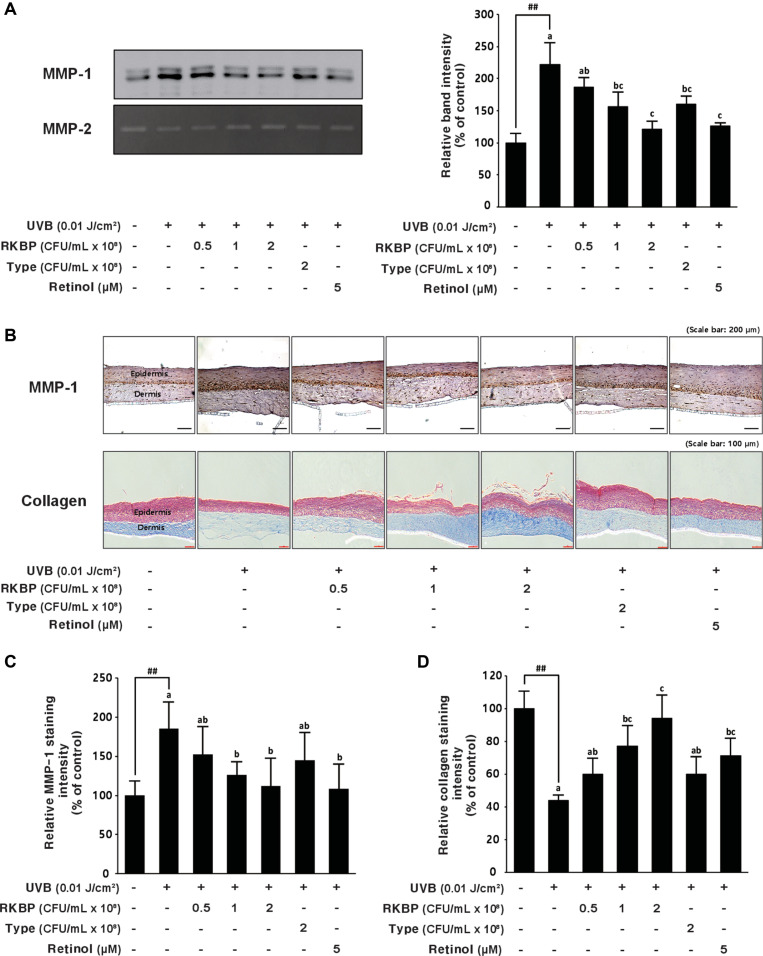
*L. plantarum* RKBP attenuates UVB-induced MMP-1 overexpression and collagen downregulation in a 3D human skin-equivalent model. (**A**) Representative western blots of MMP-1 after UVB irradiation and treatment with *L. plantarum* RKBP, ATCC 14917, or retinol. MMP-1 expression was analyzed by western blotting of conditioned media, and band intensity was normalized to MMP-2 gelatinolytic activity assessed by gelatin zymography. (**B**) Immunohistochemical staining for MMP-1 (brown) and Masson's trichrome staining for collagen fibers (blue). Arrows indicate the epidermis and dermis regions. Scale bars: 200 μm (MMP-1), 100 μm (collagen). (**C, D**) Quantification of MMP-1 immunostaining intensity and collagen staining density. Type indicates *L. plantarum* ATCC 14917. Data are presented as mean ± SD (n = 3). Significant differences between the untreated control and UVB-induced group (^##^*P* < 0.01). Different letters indicate statistically significant differences among the treatment groups based on one-way ANOVA followed by Tukey's HSD test (*P* < 0.05).

To further validate the anti-photoaging effects of *L. plantarum* RKBP, a 3D human skin-equivalent model was used to assess UVB-induced MMP-1 expression and collagen downregulation. UVB irradiation markedly increased MMP-1 protein levels (Fig. 4A). Treatment with *L. plantarum* RKBP attenuated UVB-induced MMP-1 upregulation in a dose-dependent manner, whereas ATCC 14917 exhibited a weaker inhibitory effect at the same concentration. Immunohistochemical staining showed increased MMP-1 expression (brown) in UVB-exposed tissues, while Masson's trichrome staining revealed substantial UVB-induced collagen loss, as indicated by reduced blue-stained collagen fibers in the dermis (Fig. 4B). Treatment with *L. plantarum* RKBP effectively suppressed MMP-1 expression and restored collagen integrity in a dose-dependent manner. At 2 × 10^8^ CFU/mL, *L. plantarum* RKBP reduced MMP-1 expression by 47% and attenuated collagen downregulation by 89%. In contrast, ATCC 14917 produced modest recovery. Quantitative analyses of MMP-1 immunostaining intensity and collagen density (Fig. 4C and 4D) further confirmed that *L. plantarum* RKBP more effectively preserved dermal structure than ATCC 14917. Collectively, these results demonstrated that *L. plantarum* RKBP effectively suppressed UVB-induced MMP-1 overexpression and attenuated collagen downregulation in a 3D human skin model, supporting its potential as a functional anti-photoaging ingredient.

### *L. plantarum* RKBP Suppresses α-MSH-Induced Melanin Production in 2D and 3D Spheroid Models

**Fig. 5:**
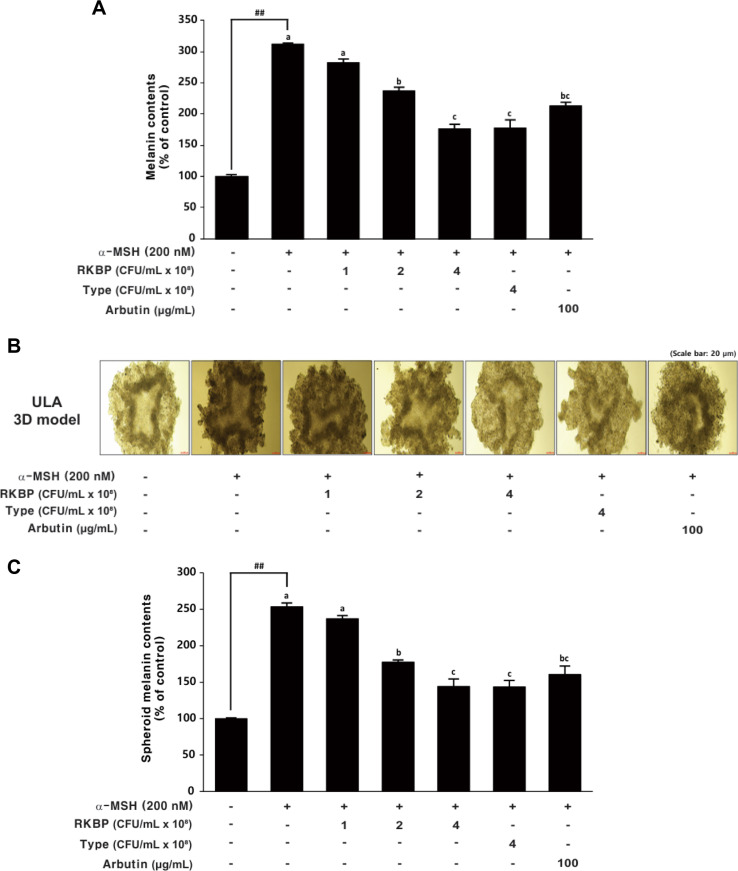
Comparative melanin inhibition by *L. plantarum* RKBP in 2D and 3D models. (**A**) Effects of *L. plantarum* RKBP on α-MSH-induced extracellular melanin levels in B16F10 cells. (**B**) Representative microscopic images of 3D spheroids in the ULA model. Lighter spheroid pigmentation indicates reduced melanin accumulation. Scale bar = 20 μm. (**C**) Extracellular melanin levels in the ULA-based 3D spheroid model were quantified by measuring the absorbance of spheroid-conditioned medium at 490 nm. Type indicates *L. plantarum* ATCC 14917. Data are presented as mean ± SD (n = 3). Significant differences between the untreated control and α-MSH-induced group (^##^*P* < 0.01). Different letters indicate statistically significant differences among the treatment groups based on one-way ANOVA followed by Tukey's HSD test (*P* < 0.05).

To evaluate the anti-melanogenic activity of *L. plantarum* RKBP, melanin content was assessed in murine B16F10 cells. α-MSH stimulation increased melanin production to approximately 3.1-fold relative to the untreated control, confirming successful induction of melanogenesis (Fig. 5A). *L. plantarum* RKBP significantly reduced α-MSH-induced melanin content in a dose-dependent manner. Specifically, *L. plantarum* RKBP at 1, 2, and 4 × 10^8^ CFU/mL decreased melanin content by 9.5%, 24%, and 43%, respectively, compared with the α-MSH-treated group. To determine whether these inhibitory effects extend to a three-dimensional environment, a 3D spheroid melanocyte model was generated using ULA plates. *L. plantarum* RKBP treatment resulted in a dose-dependent lightening of spheroid pigmentation, indicating reduced melanin accumulation (Fig. 5B). This observation was supported by quantitative analysis of extracellular melanin in spheroid-conditioned medium, which also showed reduced melanin levels following RKBP treatment (Fig. 5C). Collectively, these findings demonstrate that *L. plantarum* RKBP effectively suppresses α-MSH-induced melanin production in both 2D and 3D melanocyte models.

### *L. plantarum* RKBP Suppresses α-MSH-Induced Melanogenesis by Modulating Tyrosinase and MITF Expression

**Fig. 6:**
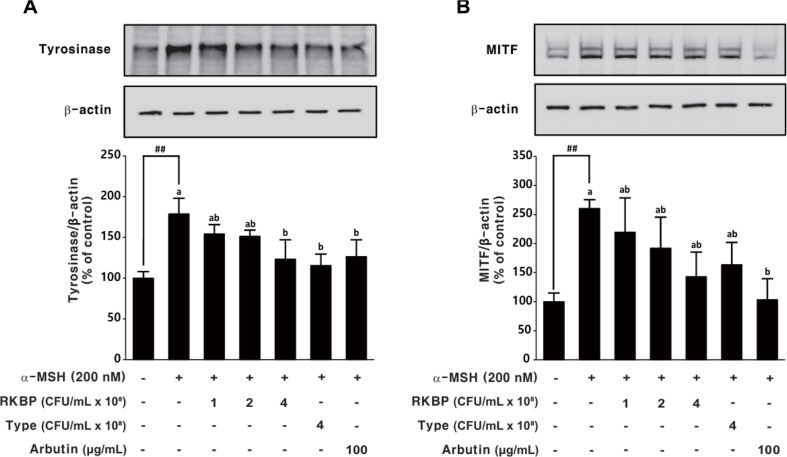
*L. plantarum* RKBP modulates the expression of tyrosinase and MITF. B16F10 cells were stimulated with α-MSH and treated with *L. plantarum* RKBP, ATCC 14917, or arbutin. (**A**) Representative western blot bands and quantification of tyrosinase expression normalized to β-actin. (**B**) Representative western blot bands and quantification of MITF expression. Type indicates *L. plantarum* ATCC 14917. Data are presented as mean ± SD (n = 3). Significant differences between the untreated control and α-MSH-induced group (^##^*P* < 0.01). Different letters indicate statistically significant differences among the treatment groups based on one-way ANOVA followed by Tukey's HSD test (*P* < 0.05).

To investigate the anti-melanogenic effects of *L. plantarum* RKBP, the expression levels of tyrosinase and MITF were analyzed in α-MSH-stimulated B16F10 cells. Tyrosinase expression was significantly reduced at 4 × 10^8^ CFU/mL, with a 30% decrease compared to the α-MSH-induced group (Fig. 6A). Meanwhile, *L. plantarum* RKBP treatment (1, 2, and 4 × 10^8^ CFU/mL) showed a tendency to reduce MITF expression in a dose-dependent manner, although the differences did not reach statistical significance compared to the α-MSH-induced group (Fig. 6B). Overall, these results suggest that *L. plantarum* RKBP may inhibit melanogenesis, particularly through suppression of tyrosinase expression.

### *L. plantarum* RKBP Inhibits Melanogenesis in a 3D Human Skin-Equivalent Model

**Fig. 7:**
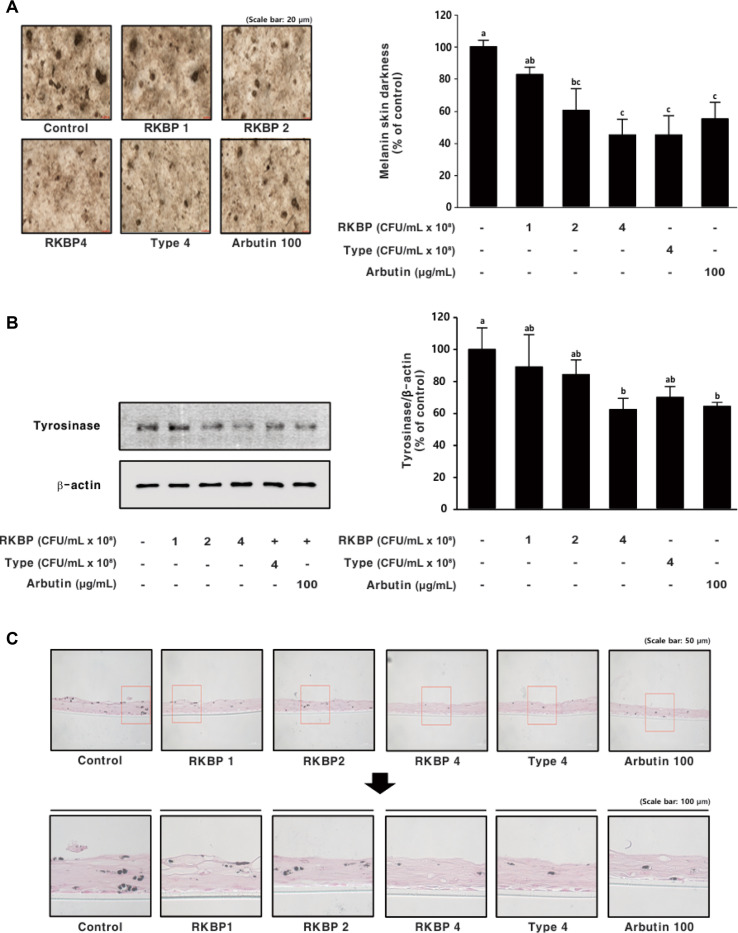
Inhibition of melanogenesis by *L. plantarum* RKBP in a 3D human skin-equivalent model. (**A**) Representative images of Neoderm^®^-ME tissues and quantification of melanin content. Scale bar = 20 μm. (**B**) Representative western blot bands and quantification of tyrosinase expression normalized to β-actin. (**C**) Fontana-Masson staining images visualizing melanin deposition in tissue sections. Scale bars: 50 μm (upper panels) and 100 μm (lower panels). Type indicates *L. plantarum* ATCC 14917. Data are presented as mean ± SD (n = 3). Different letters indicate statistically significant differences among treatment groups based on one-way ANOVA followed by Tukey's HSD test (*P* < 0.05).

To further validate the anti-melanogenic effects of *L. plantarum* RKBP under physiologically relevant conditions, a 3D human skin-equivalent model (Neoderm^®^-ME) composed of human epidermal keratinocytes and melanocytes was used. This model naturally develops pigmentation over time, allowing evaluation of melanin-regulating activity. The 3D human skin-equivalent model was treated with *L. plantarum* RKBP at concentrations of 1, 2, and 4 × 10^8^ CFU/mL, alongside treatments with ATCC 14917 (4 × 10^8^ CFU/mL) and arbutin (100 μg/mL) as a positive control. After 7 days of treatment, *L. plantarum* RKBP reduced melanin content in a dose-dependent manner (Fig. 7A). The expression of tyrosinase, a key enzyme in melanin synthesis, was also markedly decreased in *L. plantarum* RKBP-treated samples (Fig. 7B). Furthermore, Fontana-Masson staining revealed a notable reduction in melanin deposition across *L. plantarum* RKBP-treated tissues (Fig. 7C). Collectively, these findings demonstrate that *L. plantarum* RKBP effectively inhibits melanogenesis in a 3D human skin-equivalent model, as evidenced by reduced melanin content, decreased tyrosinase expression, and reduced melanin deposition under physiologically relevant conditions.

## Discussion

Heat-killed probiotics (postbiotics) have gained increasing attention as functional skin ingredients due to their excellent stability and retained biological activity. Previous studies have demonstrated that heat-killed *L. plantarum* postbiotics exert direct beneficial effects on skin cells, including antioxidant activity, suppression of pro-inflammatory cytokines, and enhancement of skin barrier function [20]. Furthermore, heat-killed *L. plantarum* L-137 improved skin hydration and barrier function in a randomized, placebo-controlled clinical study [21]. Our previous study demonstrated that *L. plantarum* RKBP, isolated from GCA, exhibits favorable probiotic characteristics including superior intestinal adhesion [12]. Based on these findings, we investigated whether heat-killed *L. plantarum* RKBP could exert direct anti-photoaging and anti-melanogenic effects in skin cell and tissue models.

UVB-induced photoaging proceeds through MAPK activation, AP-1 stimulation, and MMP-1 expression and collagen loss. *L. plantarum* RKBP interrupted this cascade by selectively inhibiting ERK and JNK phosphorylation without affecting p38. This selective inhibition translated into dose-dependent suppression of AP-1 activity and MMP-1 expression, ultimately preserving collagen integrity in 3D skin models. The protective mechanism is consistent with previous studies showing that heat-killed *Lactobacillus* strains exert anti-photoaging effects through modulation of the MAPK/MMP-1 pathway [22].

Given that *L. plantarum* RKBP was isolated from GCA [7], we investigated whether *L. plantarum* RKBP itself could modulate melanogenic signaling. In this study, *L. plantarum* RKBP showed a tendency to reduce MITF expression and significantly reduced tyrosinase expression in α-MSH-stimulated B16F10 cells. Similar anti-melanogenic effects were consistently observed in both ULA-derived 3D spheroid cultures and a 3D human skin-equivalent model [24]. These findings indicate that *L. plantarum* RKBP suppresses melanogenesis primarily through downregulation of tyrosinase expression. Recent studies have reported that postbiotic fractions from various *Lactobacillus* species can reduce α-MSH-induced melanin synthesis and downregulate MITF and tyrosinase expression in B16F10 cells [23]. Our results are consistent with these findings and further extend them by demonstrating similar effects in physiologically relevant 3D skin models.

The superior efficacy of *L. plantarum* RKBP compared to the reference strain (ATCC 14917) may be attributed to its unique metabolite profile, cell wall components, or other bioactive constituents. Our comparative analysis of short-chain fatty acids (SCFAs) revealed distinct, strain-specific metabolic signatures; notably, propionic acid was uniquely detected in the metabolites of RKBP, while remaining absent in the type strain (data not shown). Given that propionic acid is well-documented for its potent anti-wrinkle and whitening properties, it is highly probable that this specific SCFA profile drives the enhanced dermatological benefits of RKBP. This aligns with previous reports that *L. plantarum* strains exhibit distinct antioxidant activities and therapeutic effects even under identical conditions [25, 26], underscoring the necessity of strain-level selection. Furthermore, since RKBP was isolated from GCA, a plant renowned for its rich phytochemical content, it is plausible that the strain acquired these unique bioactive properties through environmental adaptation. Future metabolomic and structural analyses will be essential to fully elucidate the molecular basis of these superior protective effects.

Conventional anti-aging and whitening agents, such as retinol and arbutin, are widely used as positive controls in dermatological research. However, their limitations highlight the need for novel functional materials. Retinol, a representative anti-aging compound, is known to induce skin irritation, erythema, and dryness, requiring careful control of concentration and frequency of use, which limits its long-term and high-dose application [27]. Arbutin, a tyrosinase inhibitor used for skin whitening, is structurally related to hydroquinone, a well-known depigmenting agent for which safety and regulatory concerns have been raised [28]. Furthermore, arbutin primarily targets tyrosinase activity, with limited effects on upstream regulators such as MITF [29]. In contrast, heat-killed *L. plantarum* RKBP exhibits both anti-photoaging and anti-melanogenic activities while maintaining favorable stability as a heat-killed preparation, suggesting its potential as a multifunctional alternative to conventional agents.

The present study focused on the direct effects of heat-killed *L. plantarum* RKBP on skin cells and skin-equivalent models. However, *L. plantarum* RKBP may also exert beneficial effects on skin health via the gut-skin axis, given its favorable probiotic characteristics including superior intestinal adhesion [12]. Further in vivo studies are needed to determine whether oral administration of *L. plantarum* RKBP produces similar effects through this route. In addition, future research should identify the active components of *L. plantarum* RKBP and explore both direct skin effects and gut-skin axis-mediated mechanisms. Clinical studies will be essential to confirm the potential utility of *L. plantarum* RKBP as a functional ingredient for addressing photoaging and hyperpigmentation.

Collectively, these findings demonstrate that *L. plantarum* RKBP exerts anti-photoaging and anti-melanogenic effects through distinct molecular pathways. The suppression of the ERK/JNK-AP-1-MMP-1 cascade was associated with preservation of collagen integrity under UVB-induced conditions, while downregulation of tyrosinase expression was associated with reduced α-MSH-mediated melanogenesis. The observation of consistent effects across 2D cell cultures, 3D spheroid models, and 3D human skin-equivalent systems strengthens the translational relevance of these findings.

## Figures and Tables

**Table 1:** Primary antibodies used in this study.

Antibody	Company	Catalog No.	Dilution
MMP-1	Santa Cruz Biotechnology	sc-58377	1:1000
β-actin	Santa Cruz Biotechnology	sc-47778	1:1000
ERK	Cell Signaling Technology	#4695S	1:1000
p-ERK	Cell Signaling Technology	#9101S	1:1000
JNK	Cell Signaling Technology	#9252S	1:1000
p-JNK	Cell Signaling Technology	#4668S	1:1000
p38	Cell Signaling Technology	#9212S	1:1000
p-p38	Cell Signaling Technology	#4511S	1:1000
Tyrosinase	Abcam	ab170905	1:1000
MITF	Cell Signaling Technology	#12590S	1:1000
